# Knowledge and Perceptions of Dermatological Aesthetic Treatments Among Saudi Adults: A Cross-Sectional Study

**DOI:** 10.7759/cureus.78317

**Published:** 2025-01-31

**Authors:** Basim Alraddadi, Nawaf Albardi, Hatim Alwasidi

**Affiliations:** 1 General Practice, King Abdulaziz University, Jeddah, SAU; 2 Dermatology, King Fahad General Hospital, Medina, SAU

**Keywords:** barriers to treatment, botox, chemical peels, dermatological aesthetic treatments, healthcare education, knowledge, laser treatments, public perception, saudi arabia, social media influence

## Abstract

Background: Dermatological aesthetic treatments, such as Botox, dermal fillers, chemical peels, and laser therapies, have gained popularity around the world. In Saudi Arabia, where beauty standards are of significant cultural importance, demand for these treatments is on the rise. However, there is limited research on the general public’s knowledge and perceptions of these procedures. Understanding these factors is crucial for informed decision-making, public health education, and improving access to safe, effective treatments.

Methods: This cross-sectional study assessed the knowledge and perceptions of dermatological aesthetic treatments among 500 Saudi adults aged 18. Participants were recruited using stratified random sampling to ensure diverse representation across age groups, genders, and educational levels. A structured, self-administered questionnaire was developed based on literature review and expert input. It included sections on demographics, knowledge of treatments, perceptions of treatment safety, barriers, and influencing factors. Data were collected via online and paper-based surveys and analyzed using SPSS software (IBM Corp., Armonk, NY).

Results: Of the 500 participants, 53% (n = 265) were female, and the majority (53%, n = 265) were aged 25-44. Awareness of Botox was relatively high (55%, n = 275), but knowledge of other treatments, such as chemical peels (42%, n = 210), was lower. A significant proportion (46%, n = 230) were unaware of potential risks associated with laser treatments. Regarding safety, 56% (n = 280) of participants felt aesthetic treatments were safe when performed by qualified professionals, but 26% (n = 130) considered them inherently risky. Key barriers included safety concerns (33%, n = 165), cost (30%, n = 150), and social stigma (18%, n = 90). Social media influencers had a notable impact, with 44% (n = 220) of participants indicating they were significantly influenced by them. Future treatment intentions were higher among women (45.2%, n = 120) than men (29.8%, n = 60), and 71.3% (n = 355) of those with prior treatment experience were open to additional procedures.

Conclusion: While knowledge of dermatological aesthetic treatments is moderate among Saudi adults, significant misconceptions exist, particularly regarding treatment risks. Perceptions of safety are divided, with many participants expressing concerns about potential risks, costs, and social stigma. Social media plays a significant role in shaping public perceptions.

## Introduction

In recent years, dermatological aesthetic treatments have gained significant global popularity, particularly as non-invasive procedures become more accessible and socially acceptable. These treatments, which include Botox injections, dermal fillers, chemical peels, and laser therapies, are commonly used to enhance physical appearance, reduce signs of aging, and treat various skin conditions [1,2]. According to the International Society of Aesthetic Plastic Surgery (ISAPS), aesthetic treatments have steadily increased worldwide, with millions of individuals undergoing procedures annually. This surge is fueled by technological advancements, greater availability of skilled practitioners, and growing public awareness, partly driven by social media influencers and celebrity endorsements [2-4].

Saudi Arabia, as part of the Gulf Cooperation Council (GCC), has experienced a rapid expansion in the demand for dermatological aesthetic treatments. The country’s affluent population, the prevalence of a youthful demographic, and a growing focus on beauty standards contribute to the rising popularity of cosmetic procedures [3,5]. According to a study published by the Saudi Commission for Health Specialties, more than 1.5 million cosmetic procedures were performed annually in Saudi Arabia, with a notable increase in non-surgical treatments. The Kingdom’s cultural emphasis on aesthetics, especially among women, has positioned dermatological aesthetic procedures as a common means of self-care [5-7]. However, despite the growing demand, there is limited research on the general public’s knowledge and perception of these treatments, particularly in Saudi Arabia.

Knowledge and awareness of dermatological aesthetic treatments are crucial for informed decision-making. Inadequate knowledge may contribute to misconceptions and fears regarding treatment safety, side effects, and efficacy. Previous studies in Western countries have shown that while awareness of aesthetic treatments is relatively high, public knowledge remains limited and often marred by misunderstandings about potential risks and appropriate practitioners [7-10]. In contrast, in Saudi Arabia, there has been a lack of comprehensive research exploring the factors that influence individuals' willingness to undergo such treatments. While a few studies have examined the attitudes toward aesthetic procedures, many of these studies are focused on specific subgroups, such as women or healthcare professionals, leaving a gap in understanding the broader public’s views [5,8,9].

Understanding public perceptions is equally critical, as perceptions of treatment safety, social stigma, and cost can directly impact individuals' decisions to pursue these treatments. Studies from other regions suggest that factors such as media influence, societal pressures, and access to professional information significantly shape perceptions [5-9]. In Saudi Arabia, where traditional values coexist with modern influences, these factors may be especially pronounced, making it imperative to explore how cultural, social, and personal factors influence the public's views on dermatological aesthetic treatments.

This study aims to address these gaps by assessing the knowledge and perceptions of dermatological aesthetic treatments among Saudi adults. By exploring the general public's awareness, attitudes toward safety, and the barriers to treatment, this research seeks to provide valuable insights that can guide public health initiatives, improve patient education, and inform healthcare policy within Saudi Arabia’s evolving aesthetic medicine landscape.

## Materials and methods

Study design and participants

This cross-sectional study was conducted among 500 Saudi adults aged 18. Participants were recruited using a stratified random sampling method to ensure representation across different age groups, genders, and educational levels. Individuals with prior dermatological conditions requiring medical intervention, as well as healthcare professionals specializing in dermatology or aesthetic medicine, were excluded.

Survey instrument

A structured, self-administered questionnaire was developed based on a review of existing literature and expert input from dermatologists and public health specialists. The questionnaire comprised three sections: (1) demographics, assessing age, gender, and education level; (2) knowledge, evaluating awareness of dermatological aesthetic treatments; and (3) perception, assessing attitudes toward treatment safety, barriers, and influencing factors. The questionnaire was designed in a multiple-choice format to enhance reliability.

Validation and pilot testing

The survey underwent content validation by a panel of three dermatologists and two public health experts. A pilot study was conducted among 50 participants to assess clarity, reliability, and internal consistency. Minor modifications were made based on pilot feedback. The final questionnaire achieved a Cronbach’s alpha of 0.82, indicating high internal consistency.

Data collection

Data were collected between June 2024 and August 2024 using both online and paper-based questionnaires. Online distribution was conducted via social media platforms and email invitations, while paper-based surveys were administered in public areas such as shopping malls and community centers. All participants provided informed consent before participation.

Statistical analysis

Data were analyzed using SPSS (version 27.0, IBM Corp., Armonk, NY). Descriptive statistics were reported as frequencies and percentages (n, %) for categorical variables and means ± standard deviations (SD) for continuous variables. Differences in knowledge and perception by gender and prior treatment experience were analyzed using the chi-square test for categorical variables and the independent t-test for continuous variables. A p-value < 0.05 was considered statistically significant.

## Results

Participant characteristics

A total of 500 participants were included in the study. The majority were aged 25-44 years (n = 265, 53.0%), with a mean age of 36.8 ± 10.4 years. Female participants comprised 53.0% (n = 265). Regarding education, 190 (38.0%) held a bachelor’s degree, while 25 (5.0%) had no formal education. Previous experience with dermatological aesthetic treatments was reported by 195 participants (39.0%), with Botox (n = 88, 45.1%), laser treatments (n = 75, 38.4%), and chemical peels (n = 52, 26.7%) being the most frequently reported procedures (Table 1).

**Table 1:** Demographic characteristics of participants (N = 500)

Variable	Category	Frequency (n)	Percentage (%)
Age group	18–24	105	21.0
	25–34	140	28.0
	35–44	125	25.0
	45–54	85	17.0
	55 and above	45	9.0
Gender	Male	235	47.0
	Female	265	53.0
Education level	No formal education	25	5.0
	High school diploma	160	32.0
	Bachelor’s degree	190	38.0
	Master’s or higher	125	25.0
Undergone treatment?	Yes	195	39.0
	No	305	61.0

Knowledge of dermatological aesthetic treatments

Knowledge levels were variable among participants. While 275 (55.0%) correctly identified the primary purpose of Botox injections, only 210 (42.0%) recognized chemical peels as the most common resurfacing treatment. Misconceptions were prevalent; 230 (46.0%) were unaware that laser treatments may lead to skin burns or pigmentation changes. A majority, 270 (54.0%), correctly indicated that most dermatological aesthetic treatments require maintenance. Additionally, 320 (64.0%) correctly identified a licensed dermatologist or plastic surgeon as the appropriate provider, highlighting a gap in public awareness (Table 2).

**Table 2:** Knowledge of dermatological aesthetic treatments (N = 500) Notice the variation in responses showing that while some knowledge is widespread, misconceptions still exist.

Question	Correct answer	Correct responses (n)	Correct responses (%)
Purpose of Botox injections	Reducing wrinkles and fine lines	275	55.0
Most common resurfacing treatment	Chemical peels	210	42.0
Are treatments permanent?	Require maintenance	270	54.0
Side effects of laser treatment	Skin burns, pigmentation	230	46.0
Qualified professionals	Licensed dermatologist/plastic surgeon	320	64.0

Perception of safety and influencing factors

Perceptions of safety varied. While 280 (56.0%) believed that aesthetic treatments are safe when performed by a qualified professional, 130 (26.0%) considered them inherently risky, and 90 (18.0%) were unsure. The most frequently cited barriers to undergoing treatment included safety and side effects (n = 165, 33.0%), cost (n = 150, 30.0%), social stigma (n = 90, 18.0%), and lack of knowledge (n = 70, 14.0%) (Table 3).

**Table 3:** Perception of dermatological aesthetic treatments (N = 500)

Question	Response	Frequency (n)	Percentage (%)
Do you believe these treatments are safe?	Yes, if done by a professional	280	56.0
	No, they are risky	130	26.0
	I am not sure	90	18.0
Primary concern about undergoing treatment	Safety and side effects	165	33.0
	Cost	150	30.0
	Social stigma	90	18.0
	Lack of knowledge	70	14.0
	No concerns	25	5.0
Do social media influencers impact decisions?	Yes, significantly	220	44.0
	Somewhat	170	34.0
	No, not at all	110	22.0
Would you consider treatment in the future?	Yes	190	38.0
	No	160	32.0
	Maybe	150	30.0

Social media was a major influencing factor. A total of 220 participants (44.0%) indicated that influencers significantly impacted their perceptions, with an additional 170 (34.0%) acknowledging some degree of influence. Only 110 (22.0%) reported being unaffected by social media exposure.

Future treatment intentions

When asked about the likelihood of undergoing dermatological aesthetic treatments in the future, 190 (38.0%) expressed willingness, while 160 (32.0%) declined, and 150 (30.0%) remained uncertain. Women were more likely than men to consider treatment (120 (45.2%) vs. 70 (29.8%), p < 0.05). Among those who had previously undergone treatment, 139 (71.3%) indicated they would consider additional procedures, suggesting that prior experience influences future acceptance.

## Discussion

This study provides valuable insights into the knowledge and perceptions of dermatological aesthetic treatments among Saudi adults, highlighting both awareness gaps and barriers to treatment. Our findings suggest that while there is a moderate level of awareness about these procedures, significant misconceptions persist, and safety concerns, cost, and social stigma continue to influence treatment decisions. These results contribute to the growing body of literature on public perceptions of aesthetic medicine and offer critical guidance for healthcare professionals, policymakers, and public health advocates working to promote informed decision-making and address potential knowledge gaps.

Knowledge of dermatological aesthetic treatments

The study revealed that while 55% of participants correctly identified the primary purpose of Botox injections, awareness of other common treatments, such as chemical peels and laser treatments, was less comprehensive. Only 42% of respondents correctly identified chemical peels as a common resurfacing treatment, and nearly half (46%) were unaware of potential risks associated with laser treatments, such as skin burns or pigmentation changes. These findings echo those of similar studies conducted in Western countries, where a disparity between general awareness and in-depth understanding of aesthetic procedures has been documented [7-10]. The prevalence of misconceptions in our sample is concerning, as inadequate knowledge could hinder informed decision-making, resulting in unnecessary fears or hesitations regarding these treatments.

A critical aspect of this study is the finding that 64% of participants correctly identified licensed dermatologists or plastic surgeons as the appropriate providers for these treatments. However, the remaining 36% of respondents may have been influenced by the increasing prevalence of non-medical practitioners offering such services, which could raise concerns about treatment safety. In light of these findings, further public education is necessary to ensure that individuals understand the qualifications and credentials required for practitioners, as this is crucial to minimizing adverse outcomes and fostering confidence in the safety of these procedures [10-14].

Perception of safety and influencing factors

Perceptions of the safety of dermatological aesthetic treatments were divided, with over half of the participants (56%) acknowledging that treatments are safe when performed by qualified professionals. However, a significant portion (26%) regarded these treatments as inherently risky, and 18% remained uncertain. These findings highlight the need for a more nuanced public understanding of aesthetic treatments, as perceptions of risk may differ according to individual experiences, exposure to media portrayals, and cultural attitudes toward cosmetic procedures. Our results are consistent with studies from other regions, where safety concerns have been cited as a barrier to treatment, particularly among individuals who may have limited knowledge or direct experience with these procedures [7-11].

The barriers to treatment identified in this study - safety concerns (33%), cost (30%), social stigma (18%), and lack of knowledge (14%) - are consistent with previous research on aesthetic treatments. Safety concerns, in particular, have been a consistent barrier in studies across diverse populations. This aligns with the broader challenge in healthcare of addressing risk perceptions that are often amplified by media coverage and anecdotal experiences. Cost also emerged as a significant barrier, highlighting the financial accessibility of these treatments as a critical factor in their adoption. The influence of social stigma on treatment decisions further underscores the cultural context in which aesthetic procedures are perceived. In Saudi Arabia, where traditional values regarding appearance and beauty are intertwined with modern influences, concerns about social judgment may deter individuals from pursuing treatments despite a desire for aesthetic enhancement [10-15].

Social media influence

Our study also highlights the substantial role that social media plays in shaping public perceptions of dermatological aesthetic treatments. Forty-four percent of participants indicated that influencers significantly impacted their views, and an additional 34% acknowledged some degree of influence. This finding mirrors the growing recognition of social media’s power in influencing health behaviors, including cosmetic treatments. Social media platforms provide individuals with direct access to unregulated information, often shared by influencers or celebrities, which may not always be evidence-based [14-16]. Given the increasing reliance on social media for health information, there is a clear need for healthcare providers and public health authorities to engage in digital health literacy initiatives. These efforts could help ensure that the public receives accurate, balanced information about aesthetic treatments, empowering individuals to make informed choices based on scientific evidence rather than social media portrayals.

Future treatment intentions

Regarding future treatment intentions, 38% of participants expressed a willingness to undergo dermatological aesthetic treatments, with women being significantly more likely to consider these procedures than men. This aligns with global trends where women are more likely to seek aesthetic interventions, often driven by social pressures related to beauty standards and aging. Interestingly, individuals with prior treatment experience were more likely to express a willingness to undergo further procedures, suggesting that direct experience plays a crucial role in shaping attitudes toward these treatments. This finding underscores the importance of providing safe, effective, and positive treatment experiences, as they may lead to increased acceptance of future treatments.

Limitations

While this study provides important insights, several limitations should be noted. First, the cross-sectional design limits the ability to infer causality between knowledge, perceptions, and treatment intentions. Second, the use of self-reported data may have introduced biases, including social desirability bias, particularly when discussing sensitive topics such as cosmetic procedures. Additionally, while our sample was diverse in terms of age, gender, and education level, it may not fully represent rural or less affluent populations, who may have different attitudes toward and access to aesthetic treatments. Future studies should aim to explore these factors in more depth and assess the long-term impact of educational interventions on knowledge and perceptions.

## Conclusions

This study offers valuable insights into the knowledge and perceptions of dermatological aesthetic treatments among Saudi adults. The findings underscore the need for public health interventions aimed at improving awareness, correcting misconceptions, and addressing barriers such as cost and social stigma. Given the significant influence of social media, healthcare providers and policymakers should consider digital health literacy campaigns to promote evidence-based information about these treatments. By addressing the gaps in knowledge and perception, Saudi Arabia can enhance the safety and accessibility of dermatological aesthetic treatments, ultimately empowering individuals to make informed, confident decisions about their aesthetic care.
